# Elastography Method for Reconstruction of Nonlinear Breast Tissue Properties

**DOI:** 10.1155/2009/406854

**Published:** 2009-07-09

**Authors:** Z. G. Wang, Y. Liu, G. Wang, L. Z. Sun

**Affiliations:** ^1^Department of Civil and Environmental Engineering, University of Iowa, Iowa City, IA 52242, USA; ^2^Department of Biomedical Engineering, Indiana University-Purdue University Indianapolis (IUPUI), Indianapolis, IN 46202, USA; ^3^School of Biomedical Engineering and Sciences, Virginia Tech, Blacksburg, VA 24061, USA; ^4^Department of Civil and Environmental Engineering, University of California, Irvine, CA 92697, USA

## Abstract

Elastography is developed as a quantitative approach to imaging linear elastic properties of tissues to detect suspicious tumors. In this paper a nonlinear elastography method is introduced for reconstruction of complex breast tissue properties. The elastic parameters are estimated by optimally minimizing the difference between the computed forces and experimental measures. A nonlinear adjoint method is derived to calculate the gradient of the objective function, which significantly enhances the numerical efficiency and stability. Simulations are conducted on a three-dimensional heterogeneous breast phantom extracting from real imaging including fatty tissue, glandular tissue, and tumors. An exponential-form of nonlinear material model is applied. The effect of noise is taken into account. Results demonstrate that the proposed nonlinear method opens the door toward nonlinear elastography and provides guidelines for future development and clinical application in breast cancer study.

## 1. Introduction

Breast cancer is one of the major threats to public health all over the world. Currently, X-ray mammography is the primary method for early detection and characterization of breast tumors [1]. While more efficient in detecting malignancies as age increases or the breast becomes fatty, mammography fails to detect small cancers in dense breasts. Further, mammography may not be specific in terms of tumor benignity and malignancy [2–4]. 

To solve these problems associated with mammography, a number of technologies have been explored. Detection and characterization of breast tumors can be enhanced by recognizing the difference of elastic modulus (stiffness) among normal soft tissues and malignant and benign tumors. Elastic properties of breast tissues may become an indicator of histological diagnosis [5]. An imaging technology called elastography was developed as an approach to imaging tissue elastic modulus in a quantitative manner for detection of breast tumors in 1990s. The general basis of elastography is to induce motion within tissues under investigation by either an external or internal mechanical stimulation. Conventional medical imaging modalities are then used to measure the spatial deformation, from which the mechanical properties can subsequently be reconstructed. Most simulations are either ultrasound or magnetic resonance (MR) image-based [6–10] that take the dynamic or quasistatic interior displacement field, completely or partially, as input for identification of the elasticity properties. Current elastography reconstruction framework is based on the assumption of linear elasticity theory. It is shown, however, that the deformation of most biological soft tissues are not linearly elastic [5, 11]. Consideration of nonlinear models is essential for elastography in clinical applications.

The present work aims at developing a nonlinear elastography model for breast tissues. We introduce a nonlinear adjoint gradient method that significantly improves the numerical efficiency and enhances the stability of elastography reconstructions. In Section 2, we present an algorithm based on nonlinear adjoint gradient method. We apply the iterative Newton method to solve unknown displacements and forces. In order to find elastic modulus distribution that minimizes the objective function based on measured and calculated forces, the adjoint gradient method is employed to provide user-supplied gradients in nonlinear elastography. Numerical simulation is described in Section 3 including establishing a three-dimensional (3D) heterogeneous FEM breast phantom and applying exponential-form nonlinear material model. Four types of compressive loadings are applied in the forward problem. The iterative reconstruction based on force measurements on surface is also detailed. In Section 4, the result of the inverse problem is obtained and the effect of noise is investigated. A user-defined penalty function is introduced to reduce the impact of noise on reconstruction. 

## 2. Methodology

### 2.1. Finite-Strain Deformation Equations

We use continuum description for the breast tissues. Let Ω^0^ be a biological object. From the displacement tensor **u**(**X**) based on Lagragian coordinate system **X**, the deformation gradient is **F** = **I** + ∂**u**/∂**X** and the Green strain is **E** = (**F** ^*T*^ · **F** − **I**)/2 where “·” denotes the contraction operation between two tensors. The breast tissues are assumed hyperelastic so that the second Piola-Kirchhoff stress is **S** = ∂*W*(**E**; **p**)/∂**E**, where *W* is the strain energy and **p** denotes material parameters in the model. We use “;” to separate material parameters from deformation variables. The governing equation and boundary conditions for **u** are

(1) $$\begin{array}{c} \nabla \cdot \left(S\cdot F^{T}\right)+b\left(X,u\left(X\right)\right)=0\;X\in \Omega^{0},\\ N\left(X\right)\cdot \left(S\cdot F^{T}\right)=t\left(X,u\left(X\right)\right)\;X\in \Gamma_{t}^{0},\\ u\left(X\right)=\overset{̅}{u}\left(X\right)\;X\in \Gamma_{u}^{0}. \end{array}$$

The boundary of Ω^0^ consists of Γ_**t**_^0^, with **N** the outer normal, on which external force **t** is applied, and Γ_**u**_^0^ where displacement $\overset{̅}{u}$ is prescribed. Here, we consider general problems that the body force **b** and surface force **t** are deformation dependent. Following the standard finite-element (FE) method [12], the displacement **u** is discretized as nodal displacement vector {*u*} = {*u*_1_,*u*_2_}^*T*^, where *u*_2_ corresponds to $\overset{̅}{u}$ prescribed on Γ_**u**_^0^, and *u*_1_ is to be solved from nonlinear equations:

(2) $$\left\{\begin{array}{c} f_{1}^{\text{in}}\left(u_{1},u_{2};p\right)\\ f_{2}^{\text{in}}\left(u_{1},u_{2};p\right) \end{array}\right\}-\left\{\begin{array}{c} f_{1}^{\text{out}}\left(u_{1},u_{2}\right)\\ f_{2}^{\text{out}} \end{array}\right\}=\left\{\begin{array}{c} 0\\ 0 \end{array}\right\}.$$

The internal nodal force *f* ^in^ corresponds to stress **S**; that is, it changes with *u*_1_ and material parameters **p**, as *u*_2_ is given. The external nodal force *f*_1_^out^ is due to the prescribed surface force **t** on Γ_t_^0^ and body force **b** in Ω^0^. It varies with the displacement in large deformation. The nodal force *f*_2_^out^ is the unknown constraint force on Γ_**u**_^0^. A classic Quasi-Newton method is employed to solve (2) for *u*_1_ as shown in Appendix A. 

### 2.2. Nonlinear Elastography Algorithm

Experimental measurements for elastography include displacement and force. We consider that the biological object Ω^0^ is discretized into FE mesh, and the measurements are discretized consistently into nodal displacement and nodal force. We catalog the measurements as the following. (i) If the displacement at a node is known (prescribed or measured after deformation), it will be included into *u*_2_ which is considered “prescribed” in FE (2). The corresponding nodal force (known or unknown) will be in the constraint force, denoted as *F*_2_^*M*^. Note that *F*_2_^*M*^ corresponds to *f*_2_^out^ in (2). (ii) All the other nodal displacements will be in *u*_1_ and the corresponding nodal force will be in *f*_1_^out^. For category (ii), *f*_1_^out^ must be considered “prescribed” to fulfill the requirement of the well-postness of a solid-mechanics problem. 

For elastography problem, the obtained *u*_2_ and *f*_1_^out^ are known in (2), and the constraint force *F*_2_^*M*^ is considered as “measurement.” For given material parameters **p**, the unknown displacement *u*_1_ and constraint force *f*_2_^out^ (which depends on **p**) will be solved from FE (2). Elastography procedure thus searches for **p** so that the overall difference between measured *F*_2_^*M*^ and computed *f*_2_^out^ is minimum, that is, minimize objective function

(3) $$\Phi \left(\text{P}\right)={\left(f_{2}^{\text{out}}-F_{2}^{M}\right)}^{T}\Lambda \left(f_{2}^{\text{out}}-F_{2}^{M}\right),$$

where the diagonal weight matrix Λ = diag(*a*_1_, *a*_2_,…, *a*_*j*_,…). The component *a*_*j*_ = 1 when the *j*th component of *F*_2_^*M*^ is measured or *a*_*j*_ = 0 otherwise. The present algorithm is mathematically equivalent to our previous work [10] where the displacement is used as “measurement.” For breast tissue whose tangent stiffness significantly increases with strain, this “force version” shows better numerical efficiency. 

Efficient and robust optimization-based elastography schemes request user-supplied gradient ∂Φ/∂**p**. The previous elastography studies used direct or approximate finite-difference method [13, 14] for the gradient calculation. The computational expense of these methods increases proportionally with the number of material parameters, and becomes unaffordable for problems involving finite-strain deformation. Recently, an adjoint method was introduced to compute the gradient analytically [10, 15–17]. The corresponding nonlinear finite element formulas are shown in Appendix B. After *u*_1_ is solved for given **p**, the gradient is readily obtained as

(4) $$\frac{\partial \Phi }{\partial p}={\left\{\begin{array}{c} w_{1}\\ w_{2} \end{array}\right\}}^{T}\left\{\begin{array}{c} \partial f_{1}^{\text{in}}/\partial p\\ \partial f_{2}^{\text{in}}/\partial p \end{array}\right\},$$

where the virtual adjoint displacements *w*_1_ and *w*_2_ are solved from linear equations

(5) $$\begin{array}{ll} w_{2} & =2\Lambda \left(f_{2}^{\text{out}}-F_{2}^{M}\right),\\ {\left(K_{\text{eff}}\right)}^{T}w_{1} & =-{\left(\frac{\partial f_{2}^{\text{in}}}{\partial u_{1}}\right)}^{T}w_{2}, \end{array}$$

with the tangent stiffness matrix *K*_eff_ defined in (A.2). The most significant features of the adjoint method are its analytical formulation, high accuracy, and computational efficiency [18]. Since *K*_eff_ and its LU factorization have been calculated when solving the FE (2), as in (A.1), the additional expense for *w*_1_ and *w*_2_ in (5) is minimal. Furthermore, it only needs to solve one linear (5) regardless of the number of unknown parameters in **p**.

**Figure 1:**
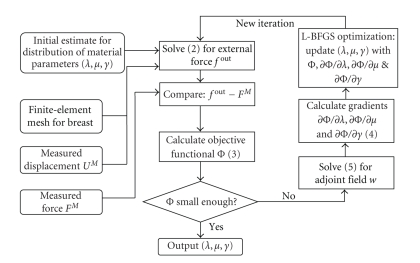
Overall flowchart for nonlinear reconstruction of material parameters *λ*, *μ*, and *γ* of breast tissues.

As shown in Figure 1, a well-tested L-BFGS subroutine is applied for the present optimization-based nonlinear elastography.

## 3. Numerical Simulations

This section presents phantom simulations to identify the nonlinear elastic properties of the fatty, glandular, and cancerous tissues in a breast. First of all, a 3D breast FEM phantom attracting from the real data is introduced. Fung's model [19] is applied to describe the deformation of breast. With the applied loading, the forward-problem computation is performed. Furthermore, boundary forces are extracted from the forward computation results and are used as input for reconstruction for a breast phantom. 

### 3.1. Breast Phantom

**Figure 2:**
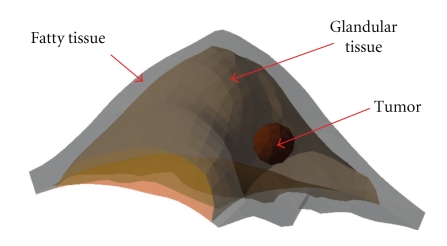
A 3D heterogeneous breast phantom extracted from real CT images, containing fatty tissue, glandular tissue, and a tumor.

**Figure 3:**
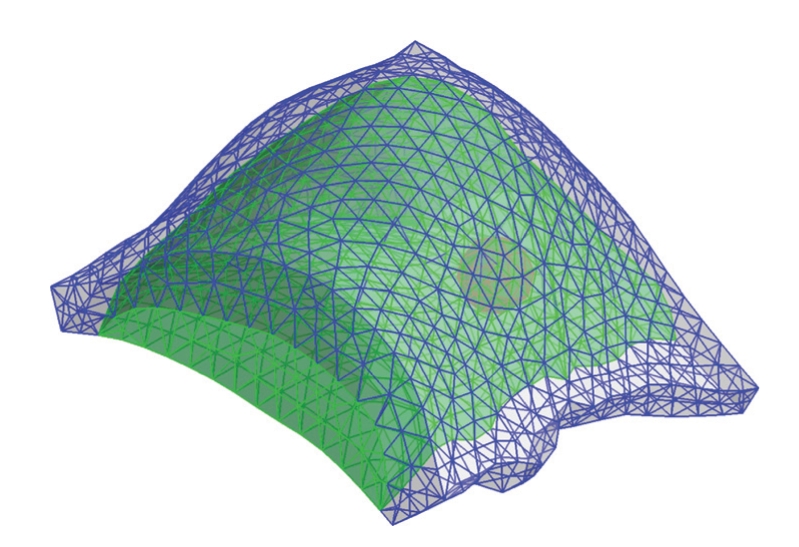
The phantom is discretized with standard 3D tetrahedral elements, consisting of 7303 elements and 1583 nodes.

To perform numerical simulations, a 3D numerical heterogeneous breast phantom extracting from real CT images, containing fatty tissue, glandular tissue, and a tumor is established (Figure 2). Boundaries of these regions are described with sets of splines. The phantom is discretized with standard 3D tetrahedral elements, consisting of 7303 elements and 1583 nodes (Figure 3).

It is well known that the mechanical behavior of biological soft tissue is nonlinear. Hyperelastic models have commonly been used to represent the stress-strain relation of biological soft tissue [20, 21]. Fung and coworkers developed a set of hyperelastic models for bio-tissues [20]. In this work, we employed a Fung-type isotropic strain-energy function for the breast tissues, as *W*(**E**;**p**) = *γ*(exp(*λ*(**E** : **I**)^2^)/2 + *μ* **E** : **E**) −1, where **E **is the Green strain, **p** = {*γ*, *λ*, *μ*} denotes the material parameters. The second Piola-Kirchhoff stress **S **is thus

(6) $$S=\frac{\partial W\left(E;p\right)}{\partial E},$$

Specifically, in uniaxial tension/compression, the relation between the axial strain *E *and axial stress *S *is

(7) $$S=\gamma \;\exp\;\left(\frac{E_{\text{Young}}E^{2}}{2}\right)E_{\text{Young}}E,$$

where *E*_Young_ = *μ*(3*λ* + 2*μ*)(*λ*+*μ*)^−1^ is the Young's modulus. 

**Figure 4:**
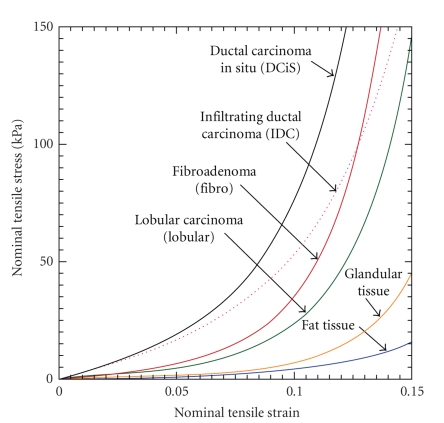
Nonlinear stress-strain curves for six breast tissues (fatty tissue, glandular tissue, lobular carcinoma, fibroadenoma, infiltrating ductal carcinoma, and ductal carcinoma in situ). (Figure redrawn based on [5] and (7).

Equation (7) is the exponentially nonlinear model that can be applied for breast tissues. Two parameters *γ* and *E*_Young_ can be determined by the experiment [22]. Wellman [5] developed a technique to measure the nonlinear elastic parameters of breast tissues using force-displacement data of thin slice tissues undergoing indentation experiment. The tissue samples tested were obtained during surgery and were tested immediately after removal from the body. Six breast tissues (fatty, gland, lobular carcinoma, fibroadenoma, infiltrating ductal carcinoma, and ductal carcinoma in situ) were tested with their nonlinear stress-train curves shown in Figure 4. It is shown that the mechanical properties between normal soft breast tissue and tumors are quite different. Tumors are stiffer than the surrounding breast tissues and malignant tumors are much stiffer than benign ones. 

In this study, exponential hyperelastic model is used and material parameters are regressed from Wellman [5] experiment. Three materials are selected: fatty tissue, glandular tissue and ductal carcinoma in situ. Elastic parameters are *λ*_*f*_ = 35, *μ*_*f*_ = 12.5, *γ*_*f*_ = 0.4 for fatty tissue, *λ*_*g*_ = 50, *μ*_*g*_ = 25, *γ*_*g*_ = 0.25 for glandular tissue and *λ*_*d*_ = 80, *μ*_*d*_ = 35, *γ*_*d*_ = 1.5 for ductal carcinoma in situ. *λ* and *μ* are dimensionless, *γ* is in kPa.

### 3.2. Forward Problem

**Figure 5:**
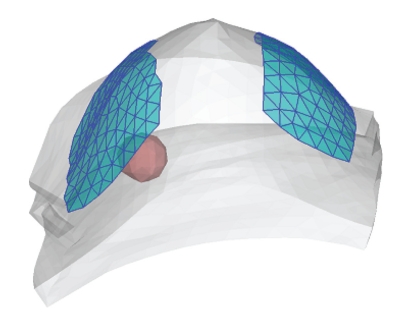
Titled compression is given by two paddles. The one close to tumor gives displacement loading on breast phantom and another is fixed during loading.

**Figure 6:**
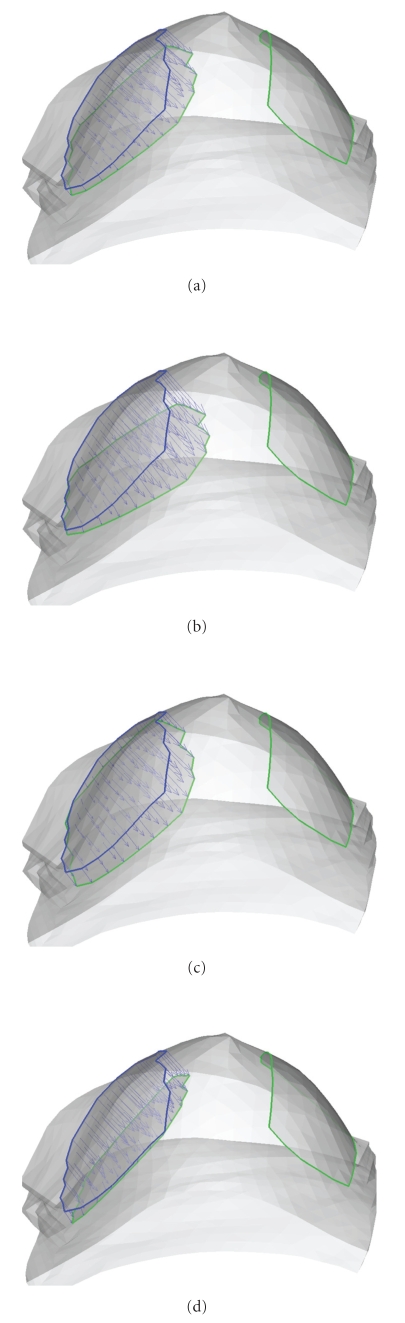
Four types of compression loading by paddles. Blue lines represent paddle locations before loadings, green lines represent after loadings. Note that the right paddle is fixed for all loadings.

**Figure 7:**
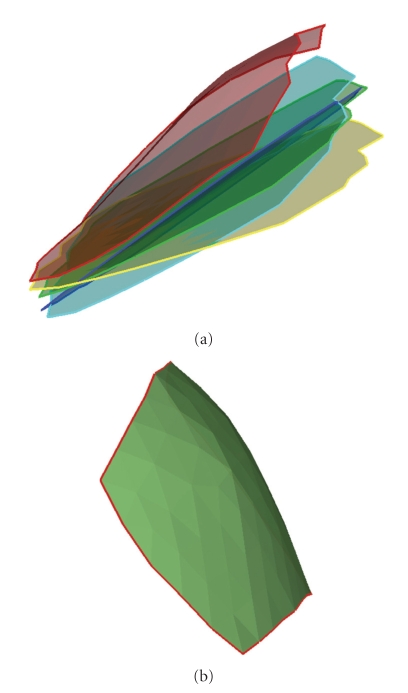
Comparison of paddle locations in four loadings. Note that the right paddle is fixed for all loadings.

After the breast phantom and material model are established, a forward problem is solved in which material parameters and external loading are given and deformation is solved. Displacements *u* = 0 are prescribed on the base of phantom. Tilted compression by paddles is applied on upper surface (Figure 5). The paddle close to tumor gives compression and another paddle is fixed during loading. Four types of compression loading with different angles are applied. As shown in Figure 6, blue lines represent paddle locations before loadings, green lines represent those after loadings. Figure 7 shows the comparison of paddle locations in four loadings. Note that the right paddle is fixed for all loadings.

The reason that four sets of loadings are applied in forward problem is to provide more information to reconstruct material parameters. Most of inverse problem in elasticity is nonuniqueness. Previous research [10] has demonstrated that one set of measurements of displacements and forces may not provide sufficient information of the reconstruction of modulus distribution. 

Given material parameters and loadings, the displacements and forces can be calculated. The surface force will be used as input to reconstruction material parameters in the inverse problem. In fact, surface displacement and force are equivalent as input to solve inverse problem. Most of previously research applied displacement in linear elastography. In this nonlinear elastography study, however, it is found the reconstruction is more sensitive to force than displacement. Therefore surface force is measured and compared with calculated force to reconstruct material parameters.

### 3.3. Inverse Problem

Reconstruction for nonlinear elastic moduli in 3D breast phantom take input extracted from the deformation in response to loading modes A ~ D described in Figure 6. In each loading, the forces on surface nodes are measured as input. Then initial guesses for material parameters are given. In this study, the same initial guesses are applied to three materials: *λ*_0_ = 20, *μ*_0_ = 10, and *γ*_0_ = 1. Therefore, the surface force can be calculated based on initial guesses of material parameters. The error between calculated and measured force is used to set up the objective function (3). Following the iterative optimization procedures (Figure 1), gradients of the objective function are calculated and material parameters are updated to approach the optimal values. 

## 4. Results and Discussion

### 4.1. Ideal Input

For three materials, elastic parameters are: *λ*_*f*_ = 35, *μ*_*f*_ = 12.5, and *γ*_*f*_ = 0.4 for fatty tissue; *λ*_*g*_ = 50, *μ*_*g*_ = 25, and *γ*_*g*_ = 0.25 for glandular tissue, and *λ*_*d*_ = 80, *μ*_*d*_ = 35, and *γ*_*d*_ = 1.5 for ductal carcinoma in situ. Table 1 shows the initial estimate and reconstructed results for nonlinear elastography. The results in the first part are based on the ideal input. It is demonstrated that the reconstructed results are very close to the real values. The maximum error is 1.916% (*λ* for tumor) since the effect of the tumor on surface force measurement is the smallest. A similar situation occurs in linear elastography reconstruction. 

### 4.2. Adjoint Methods

Elastography includes forward and inverse problem. In forward problem, material parameters and loadings are given to calculate the deformation; while in inverse problem, external loadings and deformation are known to reconstruct material parameters. Most researchers established certain objective function and minimized it with a proposed iterative algorithm. The challenge is how to calculate the gradient of objective function efficiently and accurately. A straightforward calculation of gradients requires solving stiffness matrix in each iteration, which takes most of the time consumed in the finite element method. 

In this study an adjoint method is employed to analytically calculate the gradients. Oberai et al. [16] adopted an adjoint method and proposed a numerical scheme for reconstructing the nonuniform shear modulus field for incompressible isotropic materials using one component of displacement field. Liu et al. [10] applied this method for anisotropic materials. In this study, the adjoint method is applied for nonlinear elastography. The advantage of adjoint method is to solve two adjoint displacements during each iteration (*w*_1_ and *w*_2_ at (B.2) in Appendix B), instead of the whole stiffness matrix, that increases the numerical efficiency significantly. In [18] Oberai et al. compared three different iterative methods: (1) a gradient-based method where the adjoint approach is used to calculate the gradient; (2) a gradient-based method where the straightforward approach is used to calculate the gradient, and (3) the Gauss-Newton method. The results show that “leading-order costs for the gradient-based method with the adjoint approach are smaller than the other two methods.” In fact, without the adjoint method, nonlinear elastography can only be discussed in concepts [23] or applied on simple objects using supercomputing power [24]. While in this study, material parameters in a real breast FEM phantom are reconstructed accurately by adopting this adjoint method. The results are encouraging for further clinical applications. 

### 4.3. Multiple Sets of Measurements

Above results are based on four sets of force measurements on surface. For 2D isotropic elastography, Barbone and Bamber [25] has shown that one set of measurements for the displacements and forces, especially those taken only on the boundaries, may not provide sufficient information to the reconstruction of modulus distribution, because of the nonuniqueness nature of most inverse problems in elasticity. Barbone and Gokhale [26] further demonstrated the feasibility of using multiple displacement fields to reduce the likelihood of nonuniqueness for 2D isotropic elastography. Liu et al. [10] discussed the multiple sets of measurements in 3D anisotropic media. However research effort for 3D nonlinear elastography has not been found in literatures. In this study four independent tilted compression loadings are designed and surface force is measured to reconstruct internal material parameters. Due to different initial guesses, stable material parameter can be reconstructed, showing that the multiple sets of measurements are feasible to obtain the unique solution of inverse problem in nonlinear elastography. 

The key points for using multiple sets of measurements are to bring more deformation modes simultaneously into consideration. The loadings should be close to tumors in order to make tumors have larger deformation. In Figures 5–7, four sets of loadings close to tumors are designed to obtain more deformation for reconstruction. This explanation serves as a guideline for design of loadings in clinical applications. Reduction of the number of required loadings will increase the clinical efficiency and benefit the patients. In other words, loadings with the richest stress-strain information are most desired. The design of feasible loadings is important for success in clinical application of nonlinear elastography. 

### 4.4. Input with Noise

The above results are based on ideal input. However, noise cannot be avoidable in experiments. Its impact on reconstruction is therefore investigated with 5% of noise applied on surface measurements. The results are shown in the second part of Table 1. It is obvious that the reconstructed parameters are away from the real values. A possible reason is that, while the goal is to find real material parameters that minimize the objective function, a global minimum is not well defined with noise. In another words, real material parameters may not give a global minimum of the objective function with noise. Several similar parameters around real ones may give some local minimums. Our algorithm may reach one of them but fails to find real one because they all give local minimums. A typical way to solve this problem is to add a penalty term which provides additional constraint on the solution space. The penalty term may push the solution into the right area of the solution space and minimize the resulting objective. A new objective function is therefore proposed as

(8) F=Φ+χΠ,

where Φ is the original objective function (3) which represents the difference between measured and calculated values. In addition, *χ* is the regularization factor and Π is the penalty term. 

Specific forms of penalty term have been designed for different problems [27]. In this study, an exponential form of penalty term is applied as

(9) $$\Pi =\overset{K}{\underset{k=1}{\sum }}\left(1-\exp\;\left(-{\left(\xi_{k}-a_{k}\right)}^{2}\right)\right),$$

where *K* = 9 is the total number of material parameters, and *ξ*_*k*_ and *a*_*k*_ are the reconstructed and true elastic parameters, respectively. If the true material parameters are unknown, *a*_*k*_ can be estimated as close as possible. This is reasonable since several experiments have provided the nonlinear elastic parameter for breast tissues. 

The results are shown in the third part of Table 1, demonstrating that the regularization method significantly improves the reconstruction data. Most of parameters are close enough to the true values. The largest error occurs for *χ* in fatty tissue with approximately 15%. By adding the penalty term, the reconstructed values are pulled from local minimum into the range close to true values. It is noted that the regularization factor *χ* varies in different scenarios. For example, *χ* can be smaller for higher confidence of experiment-data accuracy. On the contrary, if *a*_*k*_ is closer to the true value, *χ* should be larger. For this study, the elastic parameters in fatty and glandular tissues are stable, comparing with the ones in tumor (Figure 4). Therefore, larger regularization factors are used for fatty and glandular tissues while smaller *χ* can be applied for tumors. 

## 5. Conclusions

This paper presents a study on nonlinear elastography of biomedical imaging, in which a 3D model is developed for heterogeneous breast tissues extracting from real images including fatty tissue, glandular tissue, and tumors. Based on the large-deformation constitutive law, discretized nonlinear equations are solved for displacement, strain, and stress fields in breast tissues with given tumors under external compression at breast boundaries. A 3D inverse-problem algorithm is developed to reconstruct the material parameters for nonlinear elastic constitutive relation of breast phantoms with tumors. The adjoint gradient method is introduced to improve the numerical efficiency and enhance the stability of elastography reconstruction. Results demonstrate that this work opens the door toward nonlinear elastography, and provides guidelines for future developments and clinical applications in breast cancer study. 

## Figures and Tables

**Table 1:** Initial estimate and reconstructed results for nonlinear elastogrpahy. The results in the first part are based on ideal input (without noise). The ones in the second part are based on input with 5% noise and regularization is not used. The third part is based on input with 5% noise and regularization is applied to reduce the impact of noise. (*λ* and *μ* are dimensionless, *γ* is in kPa.)

	Fatty			Glandular			Tumor		
	*λ*_*f*_	*μ*_*f*_	*γ*_*f*_	*λ*_*g*_	*μ*_*g*_	*γ*_*g*_	*λ*_*d*_	*μ*_*d*_	*γ*_*d*_
Real	35	12.5	0.4	50	25	0.25	80	35	1.5
Guess	20	10	1	20	10	1	20	10	1

Ideal input									

Recon	34.9988	12.5004	0.3999	50.0512	24.9998	0.2498	81.5331	34.9423	1.5003

5% Noise, without regularization									

Recon	22.2753	8.4147	1.5038	56.1970	21.5414	0.2509	0.0001	41.5562	2.0010

5% Noise, with exponential form regularization									

Recon	37.56290	12.48318	0.4592	50.0014	25.0042	0.2444	80.0002	39.1539	1.5020

## Appendices

### A.

A classic quasi-Newton iterative method [28] is employed to solve (2) for *u*_1_. Let *u*_1_^(*n*)^ be the trial solution of the unknown *u*_1_ at the *n*th iterative step. An improved solution *u*_1_^(*n*+1)^ = *u*_1_^(*n*)^ + *δu*_1_ can be obtained at the next step, in which *δu*_1_ is the solution of linear equations:

(A.1) $$K_{\text{eff}}^{\left(n\right)}\delta u_{1}=f_{1}^{\text{out}}\left({u_{1}}^{\left(n\right)},u_{2}\right)-f_{1}^{\text{in}}\left({u_{1}}^{\left(n\right)},u_{2};p\right),$$

where

(A.2) $$K_{\text{eff}}^{\left(n\right)}={\frac{\partial \left(f_{1}^{\text{in}}-f_{1}^{\text{out}}\right)}{\partial u_{1}}|}_{u_{1}={u_{1}}^{\left(n\right)}}.$$

The iteration is put into effect until the residue ‖*f*_1_^out^ − *f*_1_^in^‖ is smaller than the preset criterion.

### B.

It is difficult to calculate gradient ∂Φ/∂**p** because *u*_1_ is an implicated function of **P**. Here we will derive an adjoint method for the gradient ∂Φ/∂**p** in (4). We introduce the constraint (2) into the objective function (3), and obtain a Lagrangian:

(B.1) $$L\left(u_{1},p\right)={\left(f_{2}^{\text{out}}-F_{2}^{M}\right)}^{T}\Lambda \left(f_{2}^{\text{out}}-F_{2}^{M}\right)+{\left\{\begin{array}{c} w_{1}\\ w_{2} \end{array}\right\}}^{T}\left\{\begin{array}{c} \delta f_{1}^{\text{in}}-\delta f_{1}^{\text{out}}\\ \delta f_{2}^{\text{in}}-\delta f_{2}^{\text{out}} \end{array}\right\},$$

where *w*_1_ and *w*_2_ are arbitrary virtual displacements. It is noted that variables *u*_1_ and **p** are no longer coupled. It is also noted that Φ = *L* and *δ*Φ = *δL* under the constraint (2). Since the equality constraint (2) is satisfied with *u*_1_, the variation *δL* can be expressed as

(B.2) $$\delta L=\left(2{\left(f_{2}^{\text{out}}-F_{2}^{M}\right)}^{T}\Lambda -w_{2}^{T}\right)\left(\delta f_{2}^{\text{out}}\right)+w_{1}^{T}\left(\delta f_{1}^{\text{in}}-\delta f_{1}^{\text{out}}\right)+w_{2}^{T}\delta f_{2}^{\text{in}}.$$

It can be simplified by setting the arbitrary virtual displacement *w*_2_ as

(B.3) $$w_{2}=2\Lambda \left(f_{2}^{\text{out}}-F_{2}^{M}\right),$$

so that

(B.4) $$\delta L=w_{1}^{T}\left(\delta f_{1}^{\text{in}}-\delta f_{1}^{\text{out}}\right)+w_{2}^{T}\delta f_{2}^{\text{in}}.$$

Consider that *u*_1_ and **p** are independent in *L* and that the external force term *f*_1_^out^ should not explicitly depend on the material parameter **p** and Δ*u*_2_ = 0, (B.4) becomes

(B.5) $$\delta L=\left(w_{1}^{T}\frac{\partial \left(f_{1}^{\text{in}}-f_{1}^{\text{out}}\right)}{\partial u_{1}}+w_{2}^{T}\frac{\partial f_{2}^{\text{in}}}{\partial u_{1}}\right)\delta u_{1}+\left(w_{1}^{T}\frac{\partial f_{1}^{\text{in}}}{\partial p}+w_{2}^{T}\frac{\partial f_{2}^{\text{in}}}{\partial p}\right)\delta p.$$

If we choose *w*_1_ as the solution of

(B.6) $$w_{1}^{T}\frac{\partial \left(f_{1}^{\text{in}}-f_{1}^{\text{out}}\right)}{\partial u_{1}}+w_{2}^{T}\frac{\partial f_{2}^{\text{in}}}{\partial u_{1}}=0,$$

only one term remain, that is,

(B.7) $$\delta L=\left(w_{1}^{T}\frac{\partial f_{1}^{\text{in}}}{\partial p}+w_{2}^{T}\frac{\partial f_{2}^{\text{in}}}{\partial p}\right)\delta p,$$

which yields (4) since *δ*Φ = *δL*. Equations (B.3) and (B.6) give (5) in the text.

By introducing the adjoint method, it seems that more equations and variables (*w*_1_ and *w*_2_) need to be solved. But the solution of (B.6) is straightforward and computational cost is minimized because *K*_eff_ = ∂(*f*_1_^in^ − *f*_1_^out^)/∂*u*_1_ has been computed and factorized when solving for the displacement *u*_1_ as in Appendix A. For nonlinear problem, it is very expensive to calculate ∂*u*_1_/∂**P**, while the adjoint method avoid to solved ∂*u*_1_/∂**P** by introducing variables, *w*_1_ and *w*_2_, which could be solve in simple linear equations.
